# Progress of research on PD-L1 inhibitor adebrelimab usage in malignant tumors

**DOI:** 10.3389/fonc.2024.1468569

**Published:** 2024-12-02

**Authors:** Pan Cheng, Jichen He, Pingping Cheng, Kaixia Chen, Guangyu Zhao

**Affiliations:** ^1^ Department of Pharmacy, Jingjiang People’s Hospital Affiliated to Yangzhou University, Taizhou, China; ^2^ School of Public Health, Guangxi Medical University, Nanning, China

**Keywords:** adebrelimab, programmed death-ligand 1, immune checkpoint inhibitor, extensive stage small cell lung cancer, malignant tumors

## Abstract

Adebrelimab is a humanized monoclonal antibody against programmed death-ligand 1 (PD-L1) and is also a novel immune checkpoint inhibitor, which has been used in the first-line treatment of extensive stage small cell lung cancer (SCLC) with its unique mechanism of action and good clinical efficacy. Significant progress has been made in the treatment of adebrelimab in other malignancies such as non-small cell lung cancer, triple-negative breast cancer, esophageal squamous cell carcinoma, and the treatment of SCLC at different stages is also being explored. Therefore, adebrelimab emerges as a promising new treatment option for patients with small cell lung cancer (SCLC) and other types of malignant tumors.

## 1 Introduction

Immune checkpoint inhibitors (ICIs) have become one of the remarkable breakthroughs in recent years, and their emergence has brought a new dawn for cancer treatment. It works mainly by blocking a class of proteins called immune checkpoints to restore the ability of the immune system to kill tumor cells (1). Inhibitors targeting programmed death-1 (PD-1), programmed death-ligand 1 (PD-L1) and cytotoxic T lymphocyte-associated antigen-4 (CTLA-4) have been successfully approved for the clinical treatment of various malignant tumors such as malignant lymphoma and non-small cell lung cancer (2–4). However, with the widespread use of ICIs, numerous patients experience varying degrees of immune related adverse events (irAEs), and even some patients interrupt treatment due to severe adverse reactions. Therefore, it is urgent to develop an immune checkpoint inhibitor with better efficacy and higher safety to break the dilemma for patients with malignant tumors. Adebrelimab is a PD-L1 monoclonal antibody using immunoglobulin (lg) G4 subtype immunoglobulin with superior antitumor activity and safety profile (5). In February 2023, it was approved in China for the first-line treatment of extensive stage small cell lung cancer (ES-SCLC). According to the Chinese Medical Association guideline for clinical diagnosis and treatment of lung cancer (2023 edition) for ES-SCLC, the preferred upfront treatment is the adebrelimab combined with etoposide and carboplatin regimen, as it has demonstrated excellent efficacy compared with simple chemotherapy (6). In this review, we will expound the research progress of adebrelimab in malignant tumors.

## 2 Pharmacological properties of adebrelimab

### 2.1 Pharmacodynamics

**Figure 1:**
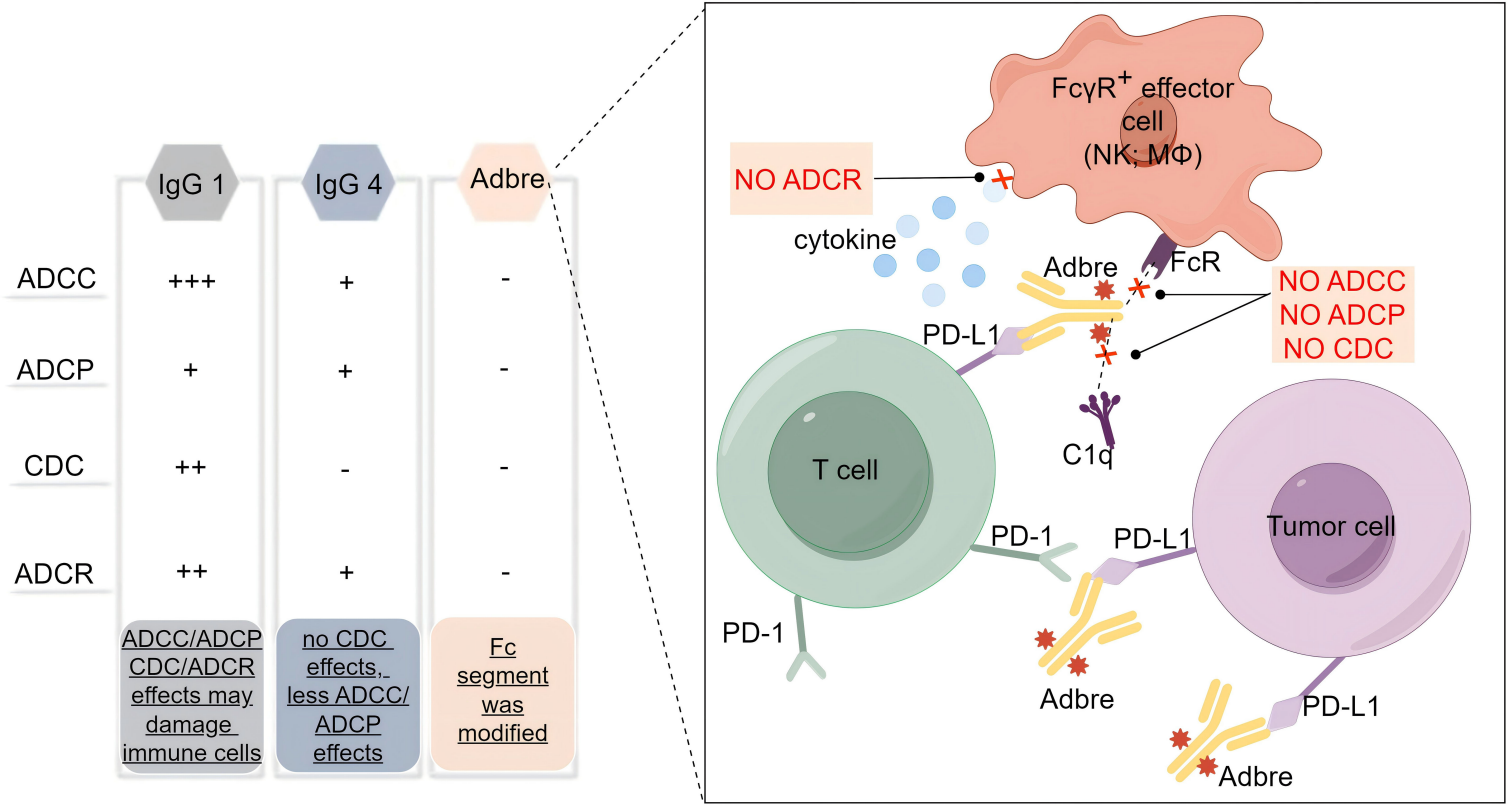
Subtype selection and structural modification of adebrelimab. Adbre, Adebrelimab; FcR, Fc receptor; FcγR, Fc gamma receptors; MΦ, macrophages; C1q, component 1q.

With the deepening understanding of the tumor immune microenvironment, it has been found that PD-L1 is not only expressed on the surface of tumor cells, but also on the surface of T cells, dendritic cells, macrophages and other immune cells, and even the expression level on the surface of some immune cells is much higher than that of tumor cells (7). This poses a challenge for the application of antibody drugs with Fc segment effector function, as the retained Fc segment effector function may not only bind and accidentally damage immune cells, but also cause uncontrollable loss of efficacy and adverse effects. The so-called Fc segment effector function refers to the hydrolysis of Ig molecules into Fab segments and Fc segments, which mainly act as antibody-dependent cell-mediated cytotoxicity (ADCC) effects, complement-dependent cytotoxicity (CDC) effects, antibody-dependent cell-mediated phagocytosis (ADCP) and antibody-dependent cytokine release (ADCR) effects. Based on the pursuit of reliable efficacy and excellent safety, the researchers developed adebrelimab to screen an ICI with purer function, higher affinity and focus on blocking the PD-L1/PD-1 signaling pathway. Therefore, adebrelimab differs from previously marketed PD-L1 inhibitors in the following aspects. Firstly, the antibody subtype selection is different (**Figure 1**). Adebrelimab uses the IgG4 antibody subtype, which has no CDC effects and is associated with less ADCC and less ADCP effects than IgG1 (8). Secondly, the antibody Fc segment was modified. Further site-directed mutagenesis modification of 234A/235A in the Fc segment of adebrelimab reduced the ability to bind to FcγR on the surface of immune cells, eliminated the ADCC and ADCP effects, and also reduced the ADCR effects (9). Meanwhile, adebrelimab Fab segment was modified by S228P to avoid Fab segment replacement and improve the stable type of the drug. Finally, the molecular structure and pharmacological properties are different. Compared with previously marketed PD-L1 antibodies, adebrelimab has a unique binding epitope to PD-L1, with a more centered binding angle and a binding region closer to the binding area of the native ligand PD-1. Furthermore, adebrelimab exhibits a dissociation constant with PD-L1 that boasts a K_d_ value of merely 0.27*10^-10^ nmol/L, indicating a stronger affinity than both durvalumab and atezolizumab, which have K_d_ values of 6.67*10^-10^ nmol/L and 4.0*10^-10^ nmol/L, respectively. (The lower the K_d_ value, the greater the affinity between the antibody and the antigen.) (5). To sum up, during the development process, adebrelimab reduced the occurrence of accidental injury and side effects of immune cells through the selection of antibody types and structural modification, thus making adebrelimab more focused on blocking the PD-1/PD-L1 pathway.

### 2.2 Pharmacokinetics

In a phase 1 trial of 41 patients with advanced solid tumors who had failed standard therapy or for whom no known effective therapy was available, adebrelimab demonstrated increased plasma exposure in a dose-proportional manner over the dose range of 3-20 mg/kg/q3w (10). After multiple administrations, the concentration reached steady state after 3-5 treatment cycles, with no obvious accumulation observed. No dose limiting toxicity was observed at any level and the maximum tolerated dose was not defined in this trial. Chen P, et al. aimed at exploring population pharmacokinetics of adebrelimab and suggested that distribution of simulated exposure metrics from the flat dose regimen (1200mg q3w) was similar to the marketed weight-based dosing regimen (20mg/kg q3w), supporting the alternative flat dose regimen in the clinic (11). In this study, there was no significant effect of baseline body weight, albumin levels, tumor size, neutrophil counts, and presence of anti-drug antibodies on CL of adebrelimab as per population-based analysis. The best structure of the adebrelimab popPK model was a two-compartmental, with time-varying linear CL described by the empirical time-dependent sigmoid-maximal model. The geometric mean of CL at baseline and at steady state (at 20th administration cycles) was 0.25L/day and 0.177L/day, respectively, and the volume of distribution at steady state was 4.91L. Geometric mean of the terminal half-life of adebrelimab at steady state was estimated at 19.9 days, which is similar to most of the mAbs.

## 3 Application of adebrelimab in malignant tumors

### 3.1 ES-SCLC

Immune-checkpoint inhibitors targeting PD-1 and PD-L1 showed good clinical activity in SCLC treatment. The results of the CAPSTONE-1 trial showed that adebrelimab as first-line treatment for ES-SCLC patients outperformed controls in overall survival and safety (5). In this phase 3 trial, 462 patients with ES-SCLC, were randomized to receive either adebrelimab combined with chemotherapy (*n*=230) or placebo combined with chemotherapy (*n*=232), and the results showed that adebrelimab combined with chemotherapy significantly improved overall survival compared to controls (median overall survival, mOS: 15.3 months *vs* 12.8 months) (5). The investigators used atezolizumab with first-line chemotherapy for ES-SCLC and found that at the median follow-up of 22.9 months, mOS and placebo with chemotherapy were 12.3 months and 10.3 months, respectively (12). And some other investigators treated durvalumab in first-line chemotherapy with a follow-up of more than 3 years. The results indicate that the mOS of durvalumab and placebo with chemotherapy was 12.9 months and 10.5 months, respectively (13). Furthermore, the ASTRUM-005 study administered a first-line treatment regimen consisting of serpliplimab in combination with chemotherapy to patients with ES-SCLC. The results showed that at a median follow-up of 12.3 months, the mOS of patients who received serpliplimab combined with chemotherapy and those who received placebo combined with chemotherapy were 15.4 months and 10.9 months, respectively (14). Wang BC et al. compared the survival data of adebrelimab (CAPSTONE-1 trial) with durvalumab (CASPIAN trial) and atezolizumab (IMpower133 trial) in the first-line setting of ES-SCLC patients, which result suggested that adebrelimab significantly prolonged survival outcomes against atezolizumab and durvalumab (15). Of course, further real-world evidence or head-to-head clinical trials are needed to confirm the differences between PD-L1 inhibitors in future studies. A network analysis of 10 randomized controlled trials (a total of 5544 ES-SCLC patients and 11 drug combination patterns) showed that PD-1/PD-L1 inhibitors-based combinations including adebrelimab are associated with significant improvement in both progression-free survival (PFS) and OS for treatment-naive ES-SCLC patients (16). The results of an additional meta-analysis of seven randomized clinical trials involving 3822 patients showed that adebrelimab plus chemotherapy exhibited better PFS outcomes compared to chemotherapy in first-line immuno-chemotherapies for ES-SCLC (17). A network meta-analysis including six phase 3 and three phase 2 randomized controlled trials (4037 ES-SCLC patients and 10 first-line regimens) showed that adebrelimab in combination with etoposide-platinum had a similar safety profile to standard chemotherapy (18).

Data from the phase study of the front-line treatment of adebrelimab combined with chemotherapy with sequential thoracic radiotherapy for ES-SCLC in 2024 showed that as of December 22, 2023, a total of 67 eligible patients were enrolled, of which 45 patients underwent thoracic radiotherapy as planned. The confirmed objective response rate (ORR) was 71.6%, and 3 (4.5%) patients achieved a complete response (CR). The mOS at the primary study endpoint was 21.4 months, the 1-year and 2-year OS rates were 74.1% and 39.7%, respectively, and the median PFS was 10.1 months (19). In addition, the incidence of Grade 3 treatment-related adverse events was 58.2%. This study showed that adebrelimab combined with chemotherapy sequential thoracic radiotherapy for first-line treatment of ES-SCLC showed good efficacy and controlled toxicity.

Some scholars analyzed from the perspective of cost-effectiveness and found that adebrelimab may be a cost-effective first-line treatment strategy for ES-SCLC compared with chemotherapy alone (20, 21). However, the latest research by Long Y et al. suggests that adebrelimab in combination with chemotherapy for the treatment of ES-SCLC was not economical compared with chemotherapy from the perspective of the Chinese healthcare system (22). Summarize the above studies, and the conclusions can be drawn that adebrelimab has good efficacy and safety in the field of ES-SCLC therapy but more studies are needed to prove its advantages in terms of cost-effectiveness.

### 3.2 Limited-stage small cell lung cancer

Concurrent chemoradiotherapy (cCRT) is generally considered the standard treatment for LS-SCLC, but the median survival of LS-SCLC is only 25-30 months, and there is still a huge unmet clinical needs (23). In March 2024, the European Lung Cancer Congress reported a randomized, multi-center phase 3 clinical study of adebrelimab and cCRT for LS-SCLC, and the results surprised many investigators. This clinical trial consists of two phases, where the first phase is a safety run-in study and the second phase is a randomized, controlled, double-blind phase 3 study. A total of 28 patients received adebrelimab combined with cCRT during the safety run-in period. At data cut-off (31 October 2023; final OS evaluation at a median follow-up of 29.4 months), the ORR was 92.9% (95% CI 76.5-99.1), disease control rate (DCR) was 100%, the median PFS was 17.9 months and the median OS has not yet reached (24). In the treatment mode of immunotherapy combined with radiotherapy and chemotherapy, no unexpected adverse reactions were found in the adverse reaction spectrum. Treatment related adverse events of 10% were all haematological toxicity with no treatment-related deaths. A randomized, double-blind, placebo-controlled phase of the study is ongoing to further evaluate this trial.

### 3.3 Non-small cell lung cancer

In recent years, the treatment of ICIs in NSCLC has achieved certain results, and various treatment modes are also being explored. A phase 1b study of 37 eligible patients in stage 2-3 NSCLC, 3 cycles of adebrelimab combined with nab-paclitaxel and carboplatin prior before surgery, and followed by 16 cycles of adebrelimab adjuvant treatment. The primary endpoint was major pathological response (MPR). The result displayed that pathologic remission was achieved in 19 patients out of 37 patients, 11 patients achieved a pathologic complete remission, 26 patients were in objective remission, the event-free survival rate at 12 months was 77.8% (25). In terms of safety, 100% of the patients enrolled in this study completed neoadjuvant therapy, which was well tolerated, found no new safety signals, mainly chemotherapy-related haematological toxicity, and no treatment-related deaths occurred. Therefore, the addition of adebrelimab with albumin combined with nab-paclitaxel and carboplatin as perioperative treatment results in a substantial proportion of MPR and high resectability with controlled toxicity. Other studies have found that the efficacy of neoadjuvant adebrelimab plus chemotherapy was basically comparable to that of nivolumab, and the resectability after neoadjuvant adebrelimab chemotherapy was similar to that of nivolumab (26, 27). Adebrelimab provides new options for the treatment of NSCLC, but further research is needed.

### 3.4 Triple-negative breast cancer

TNBC represents approximately 10-20% of all breast cancers, characterized by higher tumor mutation burden and more intensive tumor-infiltrating lymphocytes infiltration in tumor microenvironment and is more sensitive to ICIs than any other subtypes (28–30). TNBC is chemotherapy sensitive, and this treatment remains the standard of care despite its limited benefit (31). Recent advances with novel agents have been made for specific subgroups with PD-L1^+^ tumors (32). MUKDEN 03 study investigated the efficacy and safety of stereotactic body radiation therapy (SBRT) combined with adebrelimab and chemotherapy (nab-paclitaxel and carboplatin) as neoadjuvant treatment for TNBC. The results showed that adebrelimab combined with SBRT and chemotherapy for neoadjuvant treatment of TNBC had a pathological complete response (pCR) rate of 90%, ORR of 100% and with a better safety profile. This is the first study of immunotherapy combined with SBRT and chemotherapy in the neoadjuvant treatment of early TNBC, providing a novel and reliable treatment option for the neoadjuvant treatment of TNBC patients (33). In this study, 23.1% of patients experienced any level of irAEs, including 2 cases of hyperthyroidism and 1 case of hypothyroidism; One patient experienced treatment-related serious adverse reactions, but no radiation-related skin reactions or deaths occurred. This research is a great breakthrough in the field of TNBC radiotherapy combined with immunotherapy. It is of great significance and is expected to provide new treatment options for the early stages of this aggressive cancer, potentially conferring greater benefits upon patients.

### 3.5 Esophageal squamous cell carcinoma

ESCC is one of the common malignant tumors of the digestive tract. According to the global cancer data statistics, the incidence rate of ESCC ranks seventh, and the mortality rate ranks sixth (34). Neoadjuvant immunotherapy has achieved remarkable results in a variety of advanced solid tumors, but its efficacy and safety for locally advanced ESCC are still unclear (35, 36). NATION-1907 is the first study of neoadjuvant immune monotherapy for resectable ESCC, with participants being locally advanced resectable ESCC patients (37, 38). The primary endpoints were safety and feasibility, and secondary endpoints were pCR rate, OS, RFS, and R0 resection rate. A total of 25 patients completed two cycles of neoadjuvant therapy concurrent surgical resection. Research data shows the pCR rate of locally advanced resectable ESCC treated with adebrelimab neoadjuvant monotherapy is 8%, MPR rate is 24%, 2-year OS rate and RFS rate are 92% and 100%, respectively In this thrial 14 patients (56%, 14/25) had treatment-related adverse effects, most of which were grade CTCAE 1, with no new safety signals and no grade 3 or above adverse effects. A multicenter subphase study of 23 patients with inoperable resectable locally advanced or distant metastatic ESCC treated with adebrelimab combined with liposomal irinotecan and 5-fluorouracil every 14 days with primary endpoints of ORR, DCR, OS, and safety, 8.5 months, ORR and DCR of 52.2% and 73.9% after a median visit of 15.2 months, respectively, and mOS of 11.6 months (39).

### 3.6 Hepatocellular carcinoma and intrahepatic cholangiocarcinoma

HCC has an overall poor prognosis and is the third most common cause of tumor-related death worldwide (40). Some scholars have conducted a retrospective study to evaluate the safety and efficacy of adebrelimab and apatinib combined with HAIC-FOLFOX in patients with unresectable hepatocellular carcinoma (HCC). A total of 20 patients with unresectable HCC who received treatment with adebrelimab combined with apatinib and HAIC-FOLFOX were included in the study for analysis. The results showed that according to the RECIST v1.1 criteria, 7 patients achieved partial remission after treatment, with a confirmed ORR of 35% (7/20) and a disease control rate (DCR) of 85% (17/20). According to the mRECIST criteria, 2 patients achieved complete remission after treatment, and 12 patients achieved partial remission after treatment. The confirmed ORR was 70% (14/20) and DCR was 85% (17/20) (41). Another prospective phase 1b/2 clinical trial (CARES-310) aims to explore whether adebrelimab plus camrelizumab and apatinib will further increase the clinical benefit of advanced HCC therapy. At a subsequent data cut-of (4 February 2024), in the first dose group, 6 HCC patients with disease progression or intolerance after first-line treatment (systemic therapy ± surgical resection) were treated. Immune-related adverse effects were similar to other checkpoint inhibitors, and no deaths due to drug-related adverse events occurred (42). The above study demonstrated the good efficacy and controllable safety of adebrelimab combination in advanced HCC, requiring further research and exploration in the future to provide new therapeutic strategies for patients.

Due to the difficulty of early diagnosis of CCA, many patients have lost the opportunity of surgery at the time of diagnosis, resulting in a poor prognosis, and the 5-year OS is only 7%~20% (43). There was an ongoing open-label, single-arm, phase 2 trial evaluating the efficacy and safety of using adebrelimab in combination with CTLA-4 inhibitor IBI310 for the treatment of advanced iCCA patients. In this trial, 39 patients who had failed first or subsequent-line therapy were enrolled in the regimen of adebrelimab (IV 20mg/kg q3w) and IBI310 (IV 3mg/kg q3w) administered for 4 cycles, followed by SHR-1316 monotherapy until disease progression, unacceptable toxicity, or a maximum of 2 years after enrollment. At date cut-off, the median follow-up was 6.1 months (95%CI, 0.2-18.7), 28 patients were alive, 18 patients remained on treatment. Of 25 evaluable patients, 2 patients achieved complete response and 3 had partial response. The confirmed ORR and DCR were 20.0% and 60.0%, respectively. Notably, the ORR for patients previously treated with anti-PD-1 antibody was 16.7% (2/13). Median PFS and OS was not reached yet. Grade 3 or higher AEs occurred in 16 patients (41.0%), most commonly jaundice (15.4%), rash (10.3%), etc. The treatment of adebrelimab combined with IBI310 was tolerable and showed promising antitumor activity in iCCA refractory to standard therapy (44). Further studies are required to identify predictive/prognostic biomarkers to improve selection of patients most likely to benefit from this treatment strategy.

### 3.7 Ongoing studies with adebrelimab

Several ongoing clinical trials investigating the adjuvant treatment of adebrelimab for malignant tumors are summarised in **Table 1**. For example there is a multicenter, single-arm, open-label, phase 2 study (NCT06234007) investigating the efficacy and safety of short-course radiotherapy sequential fruquintinib, combined with adebrelimab in the total neoadjuvant therapy of locally advanced rectal cancer. Primary endpoint was CR rate (including clinical CR & pathologic CR) (45). Another an open-label, single-arm, prospective phase 2 trial will enroll 30 patients with III-IVB locally advanced head and neck squamous cell carcinoma (HNSCC) eligible for resection eligible for surgery to evaluate Neoadjuvant adebrelimab plus dalpiciclib in HPV-negative locally advanced HNSCC (46). One more open-label, single-arm, phase 2 study aims to evaluate the anti-tumor activity and safety of adebrelimab in combination with fuzuloparib in approximately 37 patients with homologous recombination-deficient-positive, recurrent platinum-resistant epithelial ovarian cancer, fallopian tube cancer or primary peritoneal cancer (hereinafter referred to as ovarian cancer) (47). Furthermore, a phase 4, prospective single-center, two-arm, double-blind clinical trial designed to include 110 patients with locally advanced gastric adenocarcinoma to accept adebrelimab/placebo combined with SOX regimen. The main efficacy indicators of this trial are pCR and the secondary efficacy indicators are R0 resection rate, safety indicators (**Table 1**). Outcomes from these ongoing trials may further change the treatment paradigm of patients with Patients with malignant tumors.

**Table 1:** Ongoing clinical study of adebrelimab treatment for malignant tumors.

Study	Phase	Title	Intervention/ Treatment	Endpoint
NCT06192186	Phase 4	The Safety and Efficacy of Adebrelimab Combined With SOX Regimen in Preoperative Neoadjuvant Therapy for Locally Advanced Gastric Adenocarcinoma Patients (ASOG-01)	Adebrelimab combined with SOX regimen	Efficacy and Safety
NCT06234007	Phase 2	Short-course Radiotherapy Followed by Fruquintinib Plus Adebrelimab and CAPOX in the Full Course Neoadjuvant Treatment of Locally Advanced Rectal Cancer: a Multicenter, Single-arm, Open-label Study (UNION PRECISION-I)	Fruquintinib, Adebrelimab, Oxaliplatin, Capecitabine	Efficacy and Safety
NCT06199271	Phase 2	Neoadjuvant Adebrelimab Plus Dalpiciclib in HPV-negative Locally Advanced Head and Neck Squamous Cell Carcinoma: A Phase II Clinical Trial	Adebrelimab and dalpiciclib	Efficacy and Safety
NCT05753826	Phase 2	A Single-arm, Exploratory Study of Adebrelimab Combined With Fuzuloparib in the Treatment of Patients With Recurrent Platinum-resistant Ovarian Cancer	Adebrelimab, Fuzuloparib	Efficacy and Safety
NCT06277791	Phase 2	A Single Arm, Exploratory Clinical Study of the Combination of Adebrelimab and TP Regimen for Neoadjuvant Therapy in Patients With Stage IVB Oral Squamous Cell Carcinoma in Clinical Practice	Adebrelimab	Efficacy and Safety
NCT06149130	Phase 2	Adebrelimab Combined With Dalpiciclib and Standard Endocrine Therapy for HR+/HER2-Advanced Breast Cancer:a Single-arm, Phase II Exploratory Clinical Study	Adebrelimab, dalpiciclib	Efficacy and Safety
NCT06320301	Phase 2	Evaluation of the Efficacy and Safety of Adebrelimab and a TKI in Combination With GEMOX in First-line Treatment of Advanced Biliary Tract Cancers (BTC): a Single-arm, Phase II Clinical Study	Adebrelimab+ GEMOX + TKI	Efficacy and Safety
NCT06475417	Phase 2	Phase II Study of Neoadjuvant Adebrelimab, Docetaxel, Oxaliplatin, and S-1 in Patients With Resectable Advanced Gastric Cancer	Adebrelimab combined with DOS	Efficacy and Safety
NCT06251492	Phase 2	Radiation and Adebrelimab in Prostate Cancer With Imaging-measurable Disease (RAPID):a Prospective, Single-arm, Phase II Clinical Study	Radiation: Stereotactic body radiotherapy; Drug: Adebrelimab	Efficacy
NCT06454448	Phase 1/Phase 2	Phase Ib/II Clinical Study of Adebrelimab in Combination With Decitabine, Albumin-bound Paclitaxel, and Gemcitabine for the First-line Treatment of Metastatic Pancreatic Cancer	Adebrelimab; decitabine	Efficacy and Safety

## 4 Safety profile of adebrelimab

Besides clinical efficacy, the safety profile is another important issue to consider in clinical settings. In the CAPSTONE-1 study, the most common irAEs were alanine aminotransferase increased (41%), alanine aminotransferase increased (35%) and hypothyroidism (12%) among which the incidence of ≥3 irAE did not exceed 1.8% (5). As can be seen from the **Table 2**, adebrelimab exhibited better safety profile when compared with durvalumab and atezolizumab in ES-SCLC. Due to the different patients enrolled in different studies, it is not scientific and rigorous to list the absolute values in the safety comparison. However, in the same study, the improvement rate of the test group compared with the control group can bring some enlightenment. Taking the three PD-L1 inhibitors in the field of ES-SCLC as an example, the overall improvement rate of immune combination chemotherapy compared with control groups were no more than 20%, among which the improvement rate of adebrelimab was lower, only 10.6%. In contrast, durvalumab and atezolizumab were 17.0% and 16.9%, respectively. These data presented here are merely for reference and not head-to-head study data. We eagerly anticipate more robust studies that will rigorously compare the safety profiles of PD-L1 inhibitors in the context of ES-SCLC.

**Table 2:** Main irAEs of PD-L1 inhibitors in ES-SCLC.

Drug/Study	Durvalumab (CASPIAN)	Atezolizumab (IMpower133)	Adebrelimab (CAPSTONE-1)
Death due to any irAEs (%)	5.3	1.5	0.9
Discontinuation due to any irAEs (%)	10.2	12.1	5.2
Increase rate of irAEs compared to the chemotherapy group (%)	17.0	16.9	10.6

irAEs, immune related adverse events.

## 5 Biomarkers

In the era of precision cancer therapy and personalized treatment, identifying effective biomarkers is crucial. RATIONALE 206 revealed that distinct gene expression profiles were evident among various subgroups of patients, indicating that potential biomarkers might vary across different subtypes of advanced lung cancer (48). Yu et al. found that patients with higher CD274 or YAP 1 expression showed longer PFS. Furthermore, patients with higher numbers of ZNF683^+^CD8^+^ T cells at baseline received more survival benefit from adebrelimab plus chemotherapy and sequential radiotherapy compared to patients with higher numbers of regulatory T cells at baseline (49). SCLC exhibits significant heterogeneity, with a multitude of factors influencing the regulation of its tumor immune microenvironment. Consequently, relying on a single marker to accurately predict treatment efficacy is challenging. The exploration of biomarkers for SCLC is still progressing, and the transcriptome and genomic biomarkers discovered in the above studies provide a direction for predicting the efficacy of SCLC immunotherapy. Future research needs to further validate the clinical value of these biomarkers and explore more effective therapeutic targets.

## 6 Conclusion

Adebrelimab, a fully humanized IgG4 monoclonal antibody, offers distinctive pharmacodynamic properties devoid of CDC and ADCC effects. It has shown remarkable clinical efficacy, a favorable safety profile, and broad therapeutic potential in the treatment of malignant tumors. This paper reviews the currently available data on the use of adebrelimab in patients with ES-CLSC. We eagerly anticipate the results from ongoing trials investigating adebrelimab in combination with other agents for various other types of cancer.

Simultaneously, we will encounter numerous problems and challenges, including the primary issue of how to precisely tailor adebrelimab to the individual characteristics of patients. Secondly, how to optimize the selection of treatment options for adebrelimab monotherapy or combination chemotherapy or anti angiogenic drugs is a hot topic for future research and exploration. The efficacy and economic evaluation of adebrelimab maintenance therapy following first or second-line treatment will also be a significant area of future inquiry. In addition, further exploration is needed to screen the advantageous population and reliable biomarkers for the treatment of adebrelimab, which will be a challenge in future research. Finally, it is imperative to further tackle the issue of acquired resistance to adebrelimab. It is expected that as an increasing number of high-quality clinical studies progress and evolve, further research findings will come to light. There are grounds to believe that the applications of adebrelimab in the treatment of malignant tumors are expected to expand, thereby enabling a greater number of patients with cancer to benefit from this therapy.
